# Global seafood consumption footprint

**DOI:** 10.1007/s13280-018-1060-9

**Published:** 2018-05-29

**Authors:** Jordi Guillen, Fabrizio Natale, Natacha Carvalho, John Casey, Johann Hofherr, Jean-Noël Druon, Gianluca Fiore, Maurizio Gibin, Antonella Zanzi, Jann Th. Martinsohn

**Affiliations:** 1 European Commission, Joint Research Centre (JRC). Unit D.02 Water and Marine Resources Unit, Sustainable Resources Directorate, Via E. Fermi 2749, 21027 Ispra, Italy; 2 European Commission, Joint Research Centre (JRC). Unit E.06 Demography, Migration and Governance, Directorate Space, Security and Migration, Via E. Fermi 2749, 21027 Ispra, Italy

**Keywords:** Aquaculture, Consumption footprint, Fish meal and fish oil, Fisheries, Multi-region input–output model

## Abstract

To ensure food security and nutritional quality for a growing world population in the face of climate change, stagnant capture fisheries production, increasing aquaculture production and competition for natural resources, countries must be accountable for what they consume rather than what they produce. To investigate the sustainability of seafood consumption, we propose a methodology to examine the impact of seafood supply chains across national boundaries: the seafood consumption footprint. The seafood consumption footprint is expressed as the biomass of domestic and imported seafood production required to satisfy national seafood consumption, and is estimated using a multi-regional input output model. Thus, we reconstruct for the first time the global fish biomass flows in national supply chains to estimate consumption footprints at the global, country and sector levels (capture fisheries, aquaculture, distribution and processing, and reduction into fishmeal and fish oil) taking into account the biomass supply from beyond national borders.

**Electronic supplementary material:**

The online version of this article (10.1007/s13280-018-1060-9) contains supplementary material, which is available to authorized users.

## Introduction

Over the past 50 years, annual global consumption of seafood^1^ products per capita has more than doubled, from almost 10 kg in 1960 to over 20 kg in 2014 (FAO 2016b). Seafood protein represents an essential nutritional component in many countries, especially where total protein intake levels are low. In 2013, seafood provided more than 3.1 billion people with at least 20 % of their intake of animal protein (FAO 2016b). Thus, capture fisheries and aquaculture make vital contributions to food security as a direct source of protein, micronutrients and indispensable fatty acids, but also indirectly via employment income for food purchases (Duarte et al. 2009; Godfray et al. 2010; Garcia and Rosenberg 2010; Kawarazuka and Béné 2010; Smith et al. 2010a).

According to the UN, today’s world population of more than 7 billion will rise to approximately 9 billion by 2030 and to 10 billion by 2050 (Gerland et al. 2014). Such rapid population growth will also give rise to a rapid increase in the global demand for additional food (Duarte et al. 2009; Godfray et al. 2010; Garcia and Rosenberg 2010; Béné et al. 2015). Such an increase in food production from sustainable capture fisheries is unlikely (Garcia and Grainger 2005). Total production of seafood by capture fisheries and aquaculture was estimated at 167 million tonnes in 2014 (FAO 2016a, b). Global landings from capture fisheries increased to reach more than 90 million tonnes in 1994 and stabilized thereafter^2^; while global aquaculture production more than doubled during the 1990 s with an annual growth of 10 %, falling to 6 % over the period 2000–2014 (FAO 2016a).

Aquaculture has recently superseded wild-capture fisheries as the main source of seafood for human consumption (FAO 2016b). While almost all aquaculture production is destined for human consumption, the proportion from capture fisheries now stands at around 78 %. In 2014, around three quarters of the global fish production not destined for direct human consumption was reduced to fishmeal and oil (FAO 2016b).

Aquaculture expansion, widely seen as a promising component for future food security, entails the transfer of seafood supply from capture fisheries to “farming” (Asche 2008). The dependency of farmed fish on feed originating from wild-capture fisheries is expressed by the fish-in and fish-out ratio (FIFO), a measure of the amount of wild-captured fish used to produce a unit of farmed fish. Since FIFO exceeds the value of one for many carnivorous aquaculture species, it has been argued that aquaculture growth is not necessarily offering a net gain in aquatic biomass supply (Naylor and Burke 2005). However, a fully controlled farming environment is often a more efficient production system than natural conditions (Naylor et al. 2000; Tacon and Metian 2008). Although many discussions concerning the sustainability of aquaculture development have focused on the carnivorous species or so-called ‘tigers of the sea’, that is production at high trophic levels (Naylor and Burke 2005), fishmeal consumption in other sectors including herbivorous species is also important (Tacon and Metian 2008). Considering the dependency of aquaculture on captured fish (used for the production of fishmeal and fish oil), the sustainability of aquaculture growth greatly depends on whether the aquaculture sector is able to mitigate this dependency and augment, rather than diminish the global availability of fish (Naylor et al. 2000; Tacon and Metian 2008; Hardy 2010). Fishmeal and fish oil also interconnects with the terrestrial food system through different pathways, which adds complexity to global food resilience considerations (Hardy 2010; Kristofersson and Anderson 2006; Chamberlain 2011). For instance, in 2009, 25 % of fishmeal production was used to feed pigs and 8% to feed poultry (Chamberlain 2011). Therefore, the discourse on the long-term sustainability of aquaculture in relation to its impact on captured seafood resources (mainly small and medium pelagics) has to be put into a global market and systemic context, considering dependencies between seafood demand, capture fisheries, aquaculture, livestock and feed industries (see for example Tacon and Metian 2009).

In addition to these interactions, the extensive trade in seafood commodities is an important consideration in any analysis of the seafood supply chain. Compared to other commodities, the proportion of globally produced seafood products that are traded internationally is very high and increasing, mostly due to globalization and the geographical discrepancy between aquaculture production (mostly in Asia) and seafood demand (mostly Europe, North America and Asia). The globalization of the seafood market makes possible to find seafood from all over the world in almost any developed country (Asche et al. 2015; Gephart and Pace 2015; Watson et al. 2015, 2016, 2017). In 2014, the share of global capture fisheries and aquaculture production entering international trade was 36% (FAO 2016b), the highest among food and agricultural commodities and for example, compares with around 10% for meat and 7% for milk and dairy products (Natale et al. 2015). While 78% of the seafood produced is exposed to international competition (Tveterås et al. 2012).

The high interactions between capture fisheries and aquaculture and the globalization of the seafood supply chain highlight the need to account for inter-industry flows and dependencies as well as international trade when assessing the long-term sustainability of the seafood supply chain.

Estimates of production flows from capture fisheries to aquaculture are given in Naylor et al. (2000) and Naylor and Burke (2005). Such estimates lack the detail needed to trace the flows back to the level of individual countries. In relation to capture fisheries, Swartz et al. (2010) estimated the likely origin of seafood consumed in major fishing nations. Watson et al. (2014) reconstructed the behaviour of the global fishing fleets and how changes in fishing patterns have affected seafood production. Both studies, even if explicit in geographical terms, focus on the supply from capture fisheries and take no account of existing interactions between capture fisheries and the aquaculture and feed sectors. Comparing wild-capture and mariculture production with trade data, Watson et al. (2015, 2016, 2017) examined the origin of seafood, confirming that seafood is increasingly sourced from farther origins. Watson et al. (2015) investigated the capacity that oceans may have to meet future seafood demand; while Watson et al. (2017) show that a significant share of long-distance catches from developed countries has been substituted by imports. These studies, focusing mostly on trade and supply from capture fisheries do not consider existing dependencies between all sectors and so are unable to identify the ultimate uses of seafood (e.g. direct human consumption).

The main data sources for global seafood biomass uses are the FAO food and commodity balance sheets. These provide detailed statistics on the use, supply and apparent consumption in each country. However, they do not reconstruct detailed biomass flows along the supply chain or trade patterns. The reported data in both the food supply balance sheets and the trade statistics do not permit the proportions of aquaculture in consumption and trade to be deduced, since the origins of products are not distinguished.

In this study, we aim to redress the above shortcomings on biomass flows within the seafood supply chain using a Multi-Region Input–Output model (MRIO). The MRIO models extend the Leontief’s input–output analysis (I/O) by accounting for international trade flows between different countries, which takes into account both the geographical decoupling between production and consumption through trade and the inter-industry dependencies (see for a review, Wiedmann 2009). The subsequent incorporation of environmental factors to the MRIO allows material resource flows and associated environmental impacts to be estimated, thereby permitting an assessment of the carbon and material footprints of individual nations (Lenzen et al. 2004; Peters and Hertwich 2008; Miller and Blair 2009; Davis and Caldeira 2010). Thus, MRIO models provide a systemic perspective of the sustainability concerns regarding the use of natural resources, holding importing countries accountable for global footprints by taking into account the interdependencies along the international supply chain and the connection between extraction of raw materials, inter-industry flows, trade and final consumption.

Here we develop an MRIO model for the world seafood supply chain with the aim of exploring the interactions between capture fisheries and aquaculture, fishmeal and trade at the global level. This entails reconstructing inter-industry flows of seafood biomass between countries, reconciling discrepancies in FAO and COMTRADE official statistics and reconciling published technical coefficients on feed use and seafood inputs for fishmeal and fish oil production. Thus, the main novelties of this study comprise:

- a measure of national footprints based on seafood consumption rather than production: the seafood consumption footprint;
- a breakdown of the consumption footprint by sector to quantify the dependencies between capture fisheries and aquaculture through fishmeal production and trade by country.

## Materials and methods

The core of MRIO models comprises matrices of technical coefficients describing inter-industrial flows in the economy of single countries and matrices of trade coefficients linking national economies to the rest of the world. Available I/O and MRIO tables do not have a sufficient level of sectoral disaggregation. Thus, we had to reconstruct and calibrate the basic technical coefficients and trade matrixes for our MRIO model. Such coefficients define flows of seafood biomass across the four main sectors of aquaculture, capture fisheries, seafood distribution and processing, and the fishmeal industry in individual countries and through worldwide trade. Our MRIO model has been developed in R (R Core Team 2014).

Conceptually, our MRIO model can be disaggregated in a supply table (Table 1) and in a use table (Table 2), following Eurostat (2008). The supply table represents how the supply of seafood products (Vector O) is fulfilled through domestic production (Matrix Q) and imports from other countries (Vector I). The use table represents how the different seafood products supplied are used by the different industries (i.e. intermediate consumption) interrelated on the basis of the technical coefficients by species (Matrix B), exported (Vector E) and consumed by final users, both for human consumption and for other uses (e.g. livestock and feed industries), in a country (Vector C).

**Table 1:** Supply table

Products	Sectors (industries)					Total supply
	Matrix Q				Vector I	Vector O
	Aquaculture	Fisheries	Fish processing & marketing	Fishmeal reduction	Imports	
Aquaculture species	*Q*_as_				*I*_as_	*O*_as_ = *Q*_as_ + *I*_as_
Fisheries species		*Q*_fs_			*I*_fs_	*O*_fs_ = *Q*_fs_ + *I*_fs_
Products for human consumption			*Q*_ps_		*I*_ps_	*O*_ps_ = *Q*_ps_ + *I*_ps_
Fishmeal and fish oil				*Q*_m_	*I*_m_	*O*_m_ = *Q*_m_ + *I*_m_

**Table 2:** Use table

Products	Intermediate consumption				Final uses		Total uses
	Matrix B				Vector E	Vector C	
	Aquaculture	Fisheries	Fish processing & marketing	Fishmeal reduction	Exports	Final Consumption	
Aquaculture species			*O*_as_ ∙ *b*_as p_		*E*_as_		*O*_as_ ∙ *b*_as p_ + *E*_as_ = *O*_as_
Fisheries species			*O*_fs_ ∙ *b*_fs p_	*O*_fs_ ∙ *b*_fs m_	*E*_fs_		*O*_fs_ ∙ *b*_fs p_ + *O*_fs_ ∙ *b*_fs m_ + *E*_fs_ = *O*_fs_
Products for human consumption					*E*_ps_	*C*_ps_	*E*_ps_ + *C*_ps_ = *O*_ps_
Fishmeal and fish oil	*O*_m_ ∙ *b*_m as_				*E*_m_	*C*_m_	*O*_m_ ∙ *b*_m as_ + *E*_m_ + *C*_m_ = *O*_m_

where *Q*_as_, *Q*_fs_, *Q*_ps_, *Q*_m_ is the production of each of the four products (a stands for aquaculture, f for fisheries, p for processed and marketed products for human consumption, and m for fishmeal and fish oil; and s represents data by species) from each of the four sectors; *I*_as_, *I*_fs_, *I*_ps_, *I*_m_ is the imports of each of the four products; *O*_as_, *O*_fs_, *O*_ps_, *O*_m_ is the total supply of each of the four products; *C*_ps_ is the direct human seafood consumption by species; *C*_m_ is the use of fishmeal and fish oil from sectors other than aquaculture; *E*_as_, *E*_fs_, *E*_ps_, *E*_m_ is the exports of each of the four products; *b*_as p_ is the technical coefficient depicting the proportion of aquaculture production by species going to fish processing and marketing for direct human consumption; *b*_fs p_ is the technical coefficient depicting the proportion of capture fisheries production by species going to fish processing and marketing for direct human consumption; *b*_fs m_ is the technical coefficient depicting the proportion of capture fisheries production by species used to produce fishmeal and fish oil^3^; *b*_m as_ is the technical coefficient depicting the proportion of fishmeal and fish oil used as feed in aquaculture production by species.^4^

Therefore, the direct human consumption of seafood in a country generates a demand for the productions of aquaculture and capture fisheries species (both domestic and imported) and already processed seafood net imports destined to direct human consumption (Eq. 1). The proportions of aquaculture and capture fisheries productions destined to direct human consumption are given respectively by the technical coefficients for each species *b*_as p_ and *b*_fs p_ (Eq. 2).

1 $$ C_{\text{ps}} = Q_{\text{ps}} + I_{\text{ps}} - E_{\text{ps}} $$

2 $$ C_{\text{ps}} = \left( {O_{\text{as}} \times b_{{{\text{as }}p}} } \right) + \left( {O_{\text{fs}} \times b_{{{\text{fs }}p}} } \right) + I_{\text{ps}} - E_{\text{ps}} $$

In turn, the domestic supply (i.e. production) of aquaculture generates an internal demand for fishmeal and fish oil according to the coefficient *b*_m as_. The domestic demand for fishmeal comprises the needs of the domestic aquaculture sector and the exogenous demand from the livestock sector, and is satisfied partly through the international market trade coefficient for fishmeal and partly by the capture fisheries sector according to the coefficient *b*_fs m_.

3 $$ O_{\text{m}} = Q_{\text{m}} + I_{\text{m}} - E_{\text{m}} $$

4 $$ C_{\text{m}} + \left( {O_{\text{m}} \times b_{{{\text{m}} {\text{as}}}} } \right) = \left( {O_{\text{fs}} \times b_{{{\text{fs}} {\text{m}}}} } \right) + I_{\text{m}} - E_{\text{m}} $$

So, capture fisheries supply of seafood is partly used for direct human consumption, partly to the production of fishmeal and fish oil, and partly exported.

5 $$ Q_{\text{fs}} + I_{\text{fs}} = \left( {O_{{{\text{fs}} }} \times b_{\text{fs m}} } \right) + \left( {O_{\text{fs }} \times b_{{{\text{fs }}p}} } \right) + E_{\text{fs}} $$

Table 3 shows the shares of production imported and exported between countries (Matrix T).

**Table 3:** Trade biomass flows between countries for each sector

Matrix T					
	Importer				
Exporter	Country 1	Country 2	…	Country *y*	Country *z*
Country 1	*t*_s 11_	*t*_s 12_		*t*_s 1 *y*_	*t*_s 1 *z*_
Country 2	*t*_s 21_	*t*_s 22_		*t*_s 2 *y*_	*t*_s 2 *z*_
…					
Country *y*	*t*_s *y*1_	*t*_s y2_		*t*_s *yy*_	*t*_s *yz*_
Country *z*	*t*_s *z*1_	*t*_s z2_		*t*_s *zy*_	*t*_s *zz*_

where *t*_s_ represents the trade coefficients depicting the amount of seafood from species *s* that is supplied in a country *z* through imports from country *y* (*t*_s yz_) and from domestic supply (*t*_s zz_). Thus, from Matrix T are obtained Vector *I* and Vector *E*. Vector *I*, reports for each country the imports by species of each of the four products from all countries, and Vector *E*, reports for each country the exports by species of each of the four products to all countries.

The data used to populate the model and estimate the technical coefficients described above were obtained from the FAO commodity balance sheets (FAO 2017), aquaculture and capture fisheries statistics (FAO 2016a), seafood commodities production statistics (FAO 2016a), from COMTRADE trade statistics (COMTRADE 2017) and from technical coefficients on the use of fishmeal in aquaculture and in the feed industry reported in the literature as summarized in Table 4. The use of fishmeal as feed for livestock was treated as exogenous to the model. It was estimated as a proportion of the number of livestock in each country assuming fixed allocations of 25% fishmeal for pig feed, 5% for chicken feed and 2% for other feed uses (Shepherd and Jackson 2013).

**Table 4:** Main data sources used in the study

Data	Data source
Aquaculture production	FAO (2016a, b)
Catches from fisheries	FAO (2016a, b)
Production of fishmeal	FAO (2016a, b)
Production of processed fish commodities	FAO (2016a, b)
Trade of fish commodities	COMTRADE (2017)
Apparent consumption of fish	FAO Food balance sheets (2017)
Coefficient for the conversion of fish commodities into live weight	EUMOFA (2015)
Livestock (pigs and chicken) production	FAOSTAT (2017)
Ratio of aquaculture production on aquafeed and economic feed conversion ratio and ratio of fishmeal and fish oil in aquafeed	Tacon and Metian (2015), Shepherd and Jackson (2013)
Proportion of fish for reduction into fishmeal and fish oil	Tacon and Metian (2015), Alder et al. (2008)

The final aggregated coefficients for the four main sectors of capture fisheries, aquaculture, fish distribution and processing, and fishmeal are derived for each species and commodity using the so-called ‘product mix approach’. Using such an approach, ‘recipes’ for production (e.g. the amount of fishmeal used in salmon farming) are fixed across countries, while country-specific coefficients are derived from the differences in the composition of production in each country (e.g. the share of salmon farmed in a given country against the total aquaculture production).

Relationships between the Harmonised System of classification used for trade statistics, the ASFIS List of Species for Fishery Statistics Purposes used for fisheries and aquaculture production data and the commodities classification used in FAO fish commodities production data, were defined according to the relational coefficients in Tables 1, 2 and 3. The 50 main species groups in the FAO International Standard Statistical Classification of Aquatic Animals and Plants (ISSCAAP) provided the common link between datasets.

However, fisheries official statistics suffer from data quality and missing data issues and consequently the overall production, trade and consumption levels did not match. The final household demand for fish (*C*_ps_) was taken directly from FAO food balance sheets. Thus, we used the Generalised RAS optimization method (Miller and Blair 2009) to balance Matrix T and Matrix B, in order to obtain the appropriate seafood supply per country.

The model comprised two large matrixes of technical (i.e. intra-industrial relations) and trade coefficients, which were solved against the exogenous demand vector to produce the supply vector. Simulations were carried out to estimate the global production required in the aquaculture, capture fisheries and fishmeal sectors to satisfy the overall global demand for seafood. For each simulation, the global production estimate was obtained by setting the consumption vector for all countries except the country of interest to zero.

The baseline scenario of the model was calibrated, through a series of iterations of the model, against the FAO statistics reported for aquaculture, capture fisheries and fishmeal production for 2011 (see for A1 calibrations of the model and Fig. S1 in electronic supplementary materials). The baseline scenario explicitly represents the flows of production and consumption biomass from capture fisheries, aquaculture and fishmeal by minimizing the differences between the FAO commodity balance sheets and the primary production statistics.

Estimates of national seafood production and consumption footprints can be obtained by summing the intermediate and final production and consumption of seafood and fishmeal for each country. The summed results of the individual country simulations matched the global simulation perfectly and their totals reproduced the baseline scenario for all countries combined. Our MRIO model directly calculates coefficients on the basis of seafood biomass expressed as live weight equivalents.

## Results

### Inter-industry flows

**Fig. 1:**
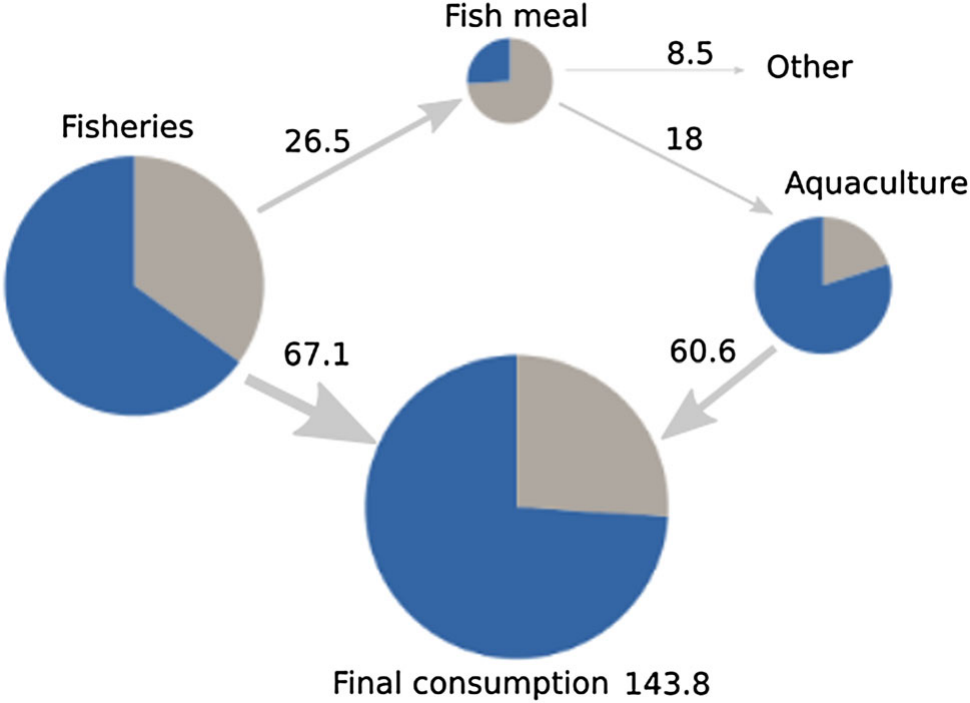
Representation of the interactions between the different sectors showing the flow of seafood products (in million tonnes) and the share of the supply with domestic (blue) or international (grey) origin for 2011

Baseline scenario results at global level are reproduced in Fig. 1. Global capture fisheries and aquaculture primary production sectors deliver 67.1 million tonnes and 60.6 million tonnes respectively to the seafood distribution and processing industries which, in turn, contribute to a global demand for seafood destined for human consumption of 143.8 million tonnes.^5^ The supply of the capture fisheries sector to the fishmeal industry is 26.5 million tonnes. Fishmeal also expressed as fish biomass live weight equivalent of 18 million tonnes is destined for the aquaculture sector and 8.5 million tonnes for other uses. While these results are broadly in agreement with the findings of Naylor and Burke (2005), we estimate lower inputs and outputs for the reduction industry, i.e. lower use of fish and lower production of fishmeal and oil.

Figure 1 indicates that 41.1% of the global capture fisheries production enters international trade. Similarly, 17.6% of global aquaculture production, 27.5% of the production from the seafood distribution and processing industries and 68.6% of the fishmeal and fish oil production are traded internationally. These results confirm the importance of international trade in seafood products; particularly the trade in fishmeal and oil and the relatively high trade in production from capture fisheries compared to aquaculture.

### Consumption versus production based footprint

**Fig. 2:**
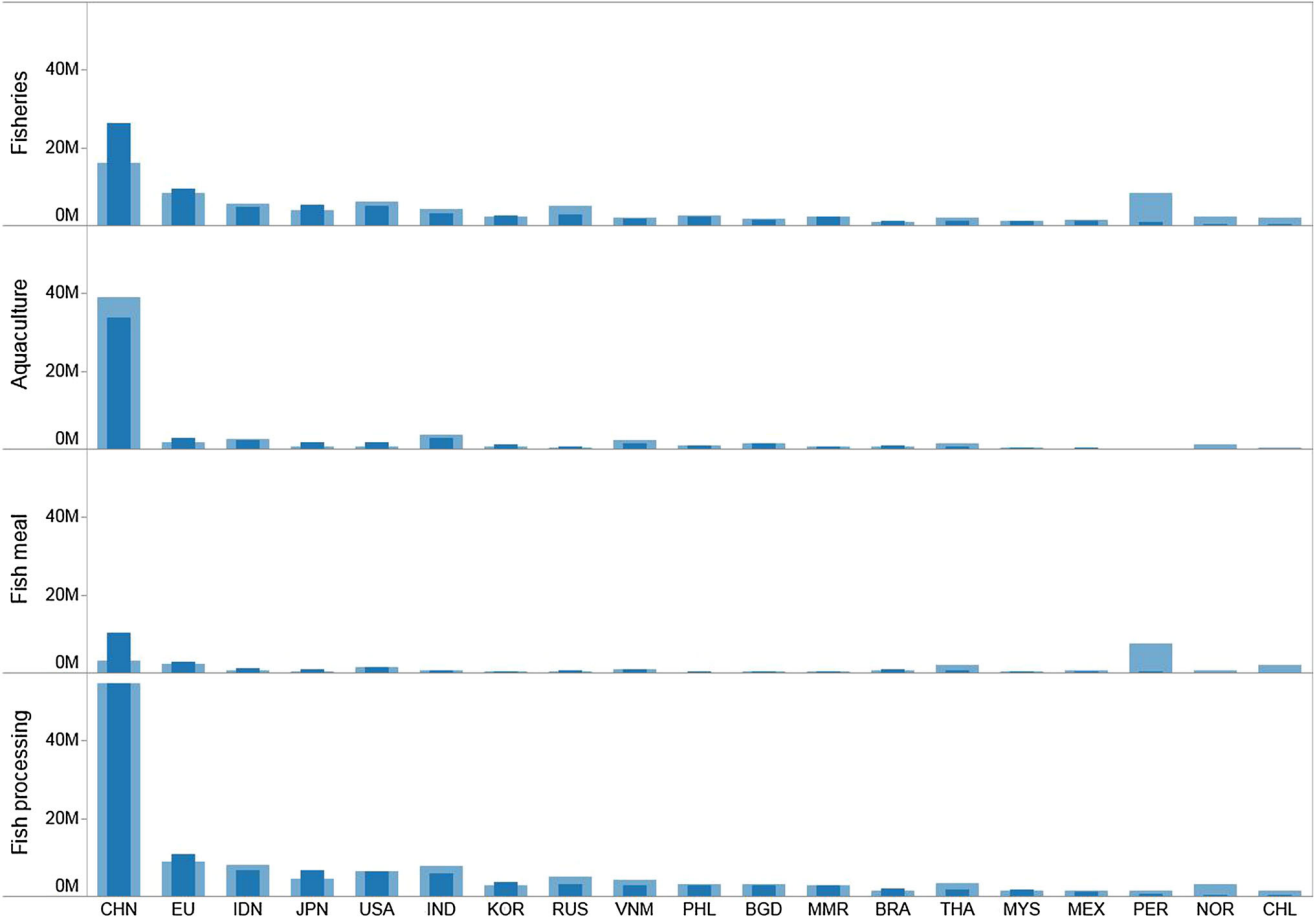
Production (light blue) and consumption (dark blue) footprint for the top 20 countries ranked according to their consumption (in million tonnes) for 2011 (note: freshwater and marine aquaculture productions are combined)

The seafood production footprint for a particular country corresponds to the production by that country and can be expressed as the proportional contribution that country makes to the global consumption, whereas the seafood consumption footprint represents the combined production by all countries that contribute to meeting the consumption of any single country. Figure 2 shows both production and consumption footprints for the top 20 countries ranked on the basis of their consumption footprint. The European Union (EU) is presented in aggregate.

What emerges from the comparison between the absolute values of production and consumption footprints is the predominant role of China both as a producer and consumer. China is largely auto-sufficient when considering the aquaculture sector alone and in this case the difference between the consumption and production footprints is small. On the contrary, China has a higher footprint as a consumer than as producer in the case of capture fisheries and fishmeal. Although aquaculture production in China is mainly based on carp species, the high consumption footprint for aquaculture creates a similarly high consumption footprint for fishmeal, due to inter-industrial linkages between the capture fisheries and aquaculture sectors. The elevated consumption footprint for fishmeal in some countries is sustained through trade, primarily with the capture fisheries and fishmeal sectors in Peru and Chile, which are the highest net producers of fishmeal.

These inter-industry flows (between capture fisheries, reduction industry and aquaculture) and international transfers are influenced by prices on the international markets for feed products (Kristofersson and Anderson 2006; Tacon and Metian 2008; Hardy 2010; Asche et al. 2013a).

### Consumption footprint by sector

**Fig. 3:**
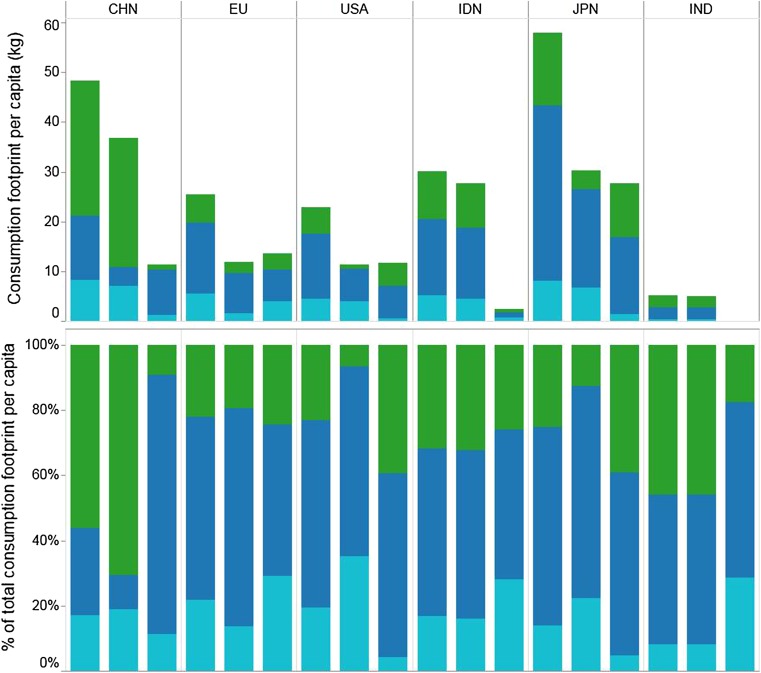
Per capita consumption footprint (kg) for the aquaculture (marine and fresh water origin, light blue), capture fisheries (dark blue) and fishmeal (green) sectors in absolute terms and as proportions (%) of the total consumption footprint for the top 6 countries ranked according to their consumption in 2011. First column of each country refers to the total per capita consumption footprint, the second one refers to the total per capita consumption footprint satisfied with domestic production and third one with external trade

Figure 3 and the Table S1 in the electronic supplementary material represent the consumption footprint per capita for the aquaculture, capture fisheries and fishmeal sectors in absolute terms and as proportions of the total consumption footprint. Results for the capture fisheries sector only include the human consumption component.

The absolute values of the consumption footprint represent both the total consumption of seafood and the preference for wild-capture or aquaculture products in the diet within each country. Compared to the FAO supply balance sheets, which also provide similar information, our model also accounts for the origin of the biomass contributing to the consumption footprint of the aquaculture and capture fisheries sectors. In addition, capture fisheries production can be accounted for separately, based on whether it is destined for direct human consumption or for the production of fishmeal.

The global per capita consumption footprint in 2011 is estimated at 27 kg. Our estimate is higher than the 18.6 kg reported in FAO statistics for the same year, as it also takes into account the indirect use of capture fisheries production by the fishmeal sector. Of the global per capita consumption footprint, 41.4% is attributable to the aquaculture sector, 41.6% to capture fisheries for direct use for human consumption and 17% to capture fisheries for indirect use through the production of fishmeal.

From the six countries with the estimated highest seafood consumption footprint, those with the highest per capita footprint are Japan with almost 60 kg, followed by China with almost 50 kg. The share of aquaculture in per capita seafood consumption is highest for China (56%) and India (46%). The disaggregation of the consumption footprint between domestic or external origin, shows that the seafood consumption in EU, USA and Japan depends significantly on imports.

## Discussion

Sustainability of seafood supply is often only assessed at the national level and generally focuses on whether the production supply from the capture fisheries and aquaculture sectors is sustainable in the long-term, taking into account biological, ecological, social and economic objectives. However, many nations rely on imports to meet national demands for seafood products. Hence, seafood sustainability assessments need to take account of domestic production and net imports which are driven by national consumption demands. Therefore, it is also important to know whether imported seafood originates from sustainable sources. Furthermore, it is important to understand the dynamics of seafood production and trade flows at a global scale in order to assess food and income security issues.

We devised a multi-region input–output model to investigate the impact of seafood consumption over national boundaries, i.e. to estimate the seafood consumption footprint. The technical and trade coefficients (Tables 1, 2) are static, reflecting the technology and trade patterns in the reference year (2011). While changes in the recipes of production embedded in the technical coefficients and in trade patterns are not expected to cause large effects in the short-term, they may have consequences in the long-term. However, potential external shocks in trade, such as the Russian trade ban on EU products imply that calibrations in the model need to be done year by year. Thus, considering that intra-sectoral relations and recipes of production should not significantly change in the short-run, the overall results for a given year are relevant and time-series analyses would not provide an increase in the added value of the study outputs. Hence, the main use of our simulations is to identify the uses and flows of seafood biomass production and consumption at a given point in time for accountability purposes, rather than for forecasting.

The key concept is that sustainability of the global seafood supply is primarily determined by the consumption demands of different nations as opposed to the seafood production within each nation. Hence, nations should be accountable for what they consume rather than for what they produce.

Such accountability implies that consumer nations hold the key to ensuring that seafood production and supply are obtained from sustainable sources. Results from our multi-region input output model can provide consumer governments with information to indicate their reliance on different producer nations for their supplies of seafood. If employed at suitable intervals and taking into account potential changes in technical coefficients and trade patterns, the changes in production and flows of seafood between nations and sectors resulting from our model can be monitored. In turn, the production sources of consumer nations can be assessed as to whether such sources are exploited in accordance the sustainability criteria and objectives set in relevant international or other legislative agreements. In cases where the relevant sustainability criteria are not met, consumer nations have the power to influence producer nations through trade agreements and by switching their sources of seafood imports. Since the collective consumption demands of different nations determine the sustainability of seafood production and supply, there is a need for such consumer nations to collaborate and cooperate to pressurize producers to take actions that are intended to meet the relevant sustainability criteria.

The seafood consumption footprint offers a clear insight into the requirements for domestic seafood production and international trade in seafood products by different sectors (capture fisheries, aquaculture, reduction and distribution and processing) to satisfy national seafood consumption demands. Sustainability of seafood consumption is therefore dependent on production beyond national borders. This is highlighted by the nature of the overall fisheries sector and the seafood market which has been highly dynamic in recent decades (Gephart and Pace 2015). The share of the total seafood products being traded internationally is very high, and it has been increasing over time. This share is the highest among food and agricultural commodities (FAO 2016b) and has been attributed largely to the effects of globalization and the disparity between the geographical distribution of aquaculture production and seafood demand. Our results (see Fig. 1) show that the share of international supply from aquaculture products is significantly lower than that from capture fisheries. Nevertheless, trade in aquaculture products has had a positive influence on trade in production from capture fisheries through development of new markets and promotion of seafood consumption in general (Valderrama and Anderson 2008).

### Prospects and challenges for seafood production and trade

Our results also confirm the high share of international trade in fishmeal and fish oil (69% enter international trade). The use of fishmeal and fish oil in competing feed industries and as alternative raw ingredients in compound feed is probably, in the global market, more driven by prices than by technological aspects. Feed represent one of the main costs for most aquaculture firms. Successive increases in fishmeal prices have caused a structural change in the capture fisheries sector leading to additional fishing pressure on low value species (Rana et al. 2009) and simultaneously an increase in the aquaculture production of omnivorous species and a reduction of feed conversion ratios (Kristofersson and Anderson 2006; Naylor et al. 2009). The aquaculture sector has tried to substitute some aquafeed inputs from capture fisheries with cheaper plant-based products (e.g. soybean meal).

Such changes have led to a decrease in the overall FIFO from 1.04 in 1995 to 0.63 or 0.52 in 2007, depending on the calculation method (Tacon and Metian 2015; Jackson 2016). That is, on average, about 1.92 tonnes of harvestable aquaculture product can be derived from every tonne of whole wild fish caught and used for feed. This is partly the result of the genetic modifications to some aquaculture species which has resulted in a more efficient use of seafood resources (Smith et al. 2010b). Furthermore, aquaculture uses seafood resources (i.e. fishmeal) more efficiently than livestock production as, for example, the feed conversion ratio^6^ for aquaculture-produced salmon (1.3) is low compared to chicken (1.9), pork (2.8) and beef (6–9) (Welch et al. 2010). Moreover, the conversion of wild-capture fish that would not be used for human consumption into fishmeal and subsequent use as aquafeed, results in an overall increase in human consumption of fish (Wijkström 2009).

Aquaculture production has become less dependent on fishmeal and oil from capture fisheries than it was in the past. However, despite such developments, the pre-2000 growth rate of global aquaculture production is showing signs of slowing down (Liu and Sumalia 2008; Asche et al. 2013b). Hence forecast indicating that aquaculture production will meet the increasing demand created by an increasing world population may be over-optimistic.

Similarly, an increase in consumption demand for animal products, such as cheap seafood products has been observed together with increases in income and purchasing power in emerging economies (e.g. China and Brazil) (Gerbens-Leenes et al. 2010). Continued increases in income and urbanization in developing countries, may lead to higher seafood prices and changes in traditional trade relations between countries. Consequently, the seafood consumption footprint in areas that currently benefit from high imports (e.g. EU, Japan and USA) may decrease. Moreover, increases in prices are likely to incentivize overfishing and consequently undermine the possibility to achieve sustainable seafood production.

## Conclusions

In this study, we advocate that the sustainability of the global seafood supply is primarily determined by the collective consumption demands of different nations. Hence, when assessing the relative national impacts on such sustainability, the domestic consumption of seafood as opposed to the domestic production, is the most suitable measure of the extent to which each nation should be held accountable. We therefore propose the “seafood consumption footprint”, which expresses domestic seafood consumption in terms of the biomass (domestic and imported) derived from the different seafood production and consumption sectors using a multi-regional input–output model. Our reconstruction of the global seafood biomass flows provides, for the first time, the proportions of national consumption originating from domestic production and from international trade by sector.

Food security and production and supply from sustainable sources, are issues high on the international political agenda and with a rapidly expanding global population, the global demand for additional food, including seafood is set to increase. The seafood consumption footprint indicates the extent to which the consumption for all nations is sourced from abroad. Such information provides national governments with evidence to encourage international collaboration and promote policies to ensure long-term sustainability of all seafood production.

## Electronic supplementary material

Below is the link to the electronic supplementary material.

Supplementary material 1 (PDF 232 kb)

## Footnotes

1 By seafood, in this study, we refer to fish, molluscs and crustaceans from capture fisheries and aquaculture, both from marine (including brackish water) and freshwater environments.

2 31% of the world's wild fish stocks are estimated to be overfished, 58% fully exploited and only 11% as under finished (FAO 2016b).

3 The total amount of fishmeal and fish oil is obtained by converting the catches of industrial species into fishmeal equivalents using the coefficient of 4.8. The estimated conversion factor of 4.8 comes from the calibration of the model. A conversion of 4.4 is often used for the conversion of whole fish to fishmeal. However, in addition to the conversion of whole fish to fishmeal, our estimate also incorporates the direct use of wild-captured fish in aquaculture and the use of trimmings from the processing sector.

4 Following Tacon and Metian (2015) and Shepherd and Jackson (2013) this amount is calculated multiplying the aquaculture production, by the feed conversion ratio, by the percentage of production using aquafeed and by the level of inclusion of fishmeal in aquafeed.

5 The 16.1 million tonnes difference (11.2% of the global consumption) could be explained by the existence of IUU fishing, under-reporting of production statistics, “seafood consumption” from inland fisheries not properly registered in consumption statistics (global inland fisheries production was 11.1 million tonnes in 2011) and potential double-counting in trade statistics.

6 Feed conversion ratio is a measure of an animal's efficiency in converting feed (in weight) into increases of the body weight.
