# Supplementary material for: Global seafood consumption footprint
Source: Ambio. 2018 May 29;48(2):111–22. doi: 10.1007/s13280-018-1060-9 (PMC6346599; doi:10.1007/s13280-018-1060-9)
Supplement: Supplementary file 1 — Supplementary material 1 (PDF 232 kb) [file 13280_2018_1060_MOESM1_ESM.pdf]

Ambio

Electronic Supplementary Material

This supplementary material has not been peer reviewed.

Title: **Global seafood consumption footprint**

Authors: Jordi Guillen, Fabrizio Natale, Natacha Carvalho, John Casey, Johann Hofherr, Jean-Noël Druon, Gianluca Fiore, Maurizio Gibin, Antonella Zanzi, Jann Th. Martinsohn.

A1: Calibration of the model

The calibrated model closely reproduces the 2011 global levels of seafood production. The differences between the model estimates and the production reported in official statistics was -2.8 million tonnes (-1.8%) for the capture fisheries and aquaculture sectors and + 62.7 thousand tonnes for the fishmeal sector (+1.1%). The estimated proportion of the total seafood production derived from aquaculture was 39.3% compared to 39.4% in the statistical data, indicating that the model was able to correctly attribute and replicate the origin and volume of the supply and trade in seafood products to the aquaculture and capture fisheries sectors.

Adjustments to the trading coefficient during calibration were mainly concerned with correcting the data for China and for other countries with large volumes of trade in transit (i.e., re-exporting of fresh and processed seafood). The need for corrections in countries of transit can be explained by the fact that the model does not capture the full trade path from true origins and destinations, resulting in an underestimation of the c_{ss} coefficients.

Despite the calibration, differences between model estimates of production and published statistical data remain in some cases (Figure S1). Such differences largely arise through errors in model estimates of production for the national capture fisheries and fishmeal sectors within specific countries, rather than to incorrect allocation of production to the aquaculture and capture fisheries sectors. Such differences are partly balanced when aggregating at the global scale.

In particular, for capture fisheries and aquaculture combined, the calibrated model estimates were lower than the production reported in the official statistics; the largest differences relate to Chile (-1.3 million tonnes), Myanmar (-1.2 million tonnes) and Taiwan (-1.2 million tonnes). In the case of fishmeal, the estimated production was lower than the official statistics for Japan (-130 thousand tonnes), Thailand (-105 thousand tonnes), Chile (-72 thousand tonnes) and Denmark (-55 thousand tonnes). These discrepancies may be attributed to the data omissions from official statistics (e.g. no data on demand and trade for Taiwan), issues related to the technical coefficients used as parameters in the model which are not able to capture country specificities or to inconsistencies between demand, trade and primary production across the different statistical data sources.

Figure S1: Calibration results for capture fisheries and aquaculture (in million tonnes) and fishmeal (thousand tonnes) for the countries showing the largest deviations based on 2011. The larger area shows the official statistic and the internal column the estimated value.

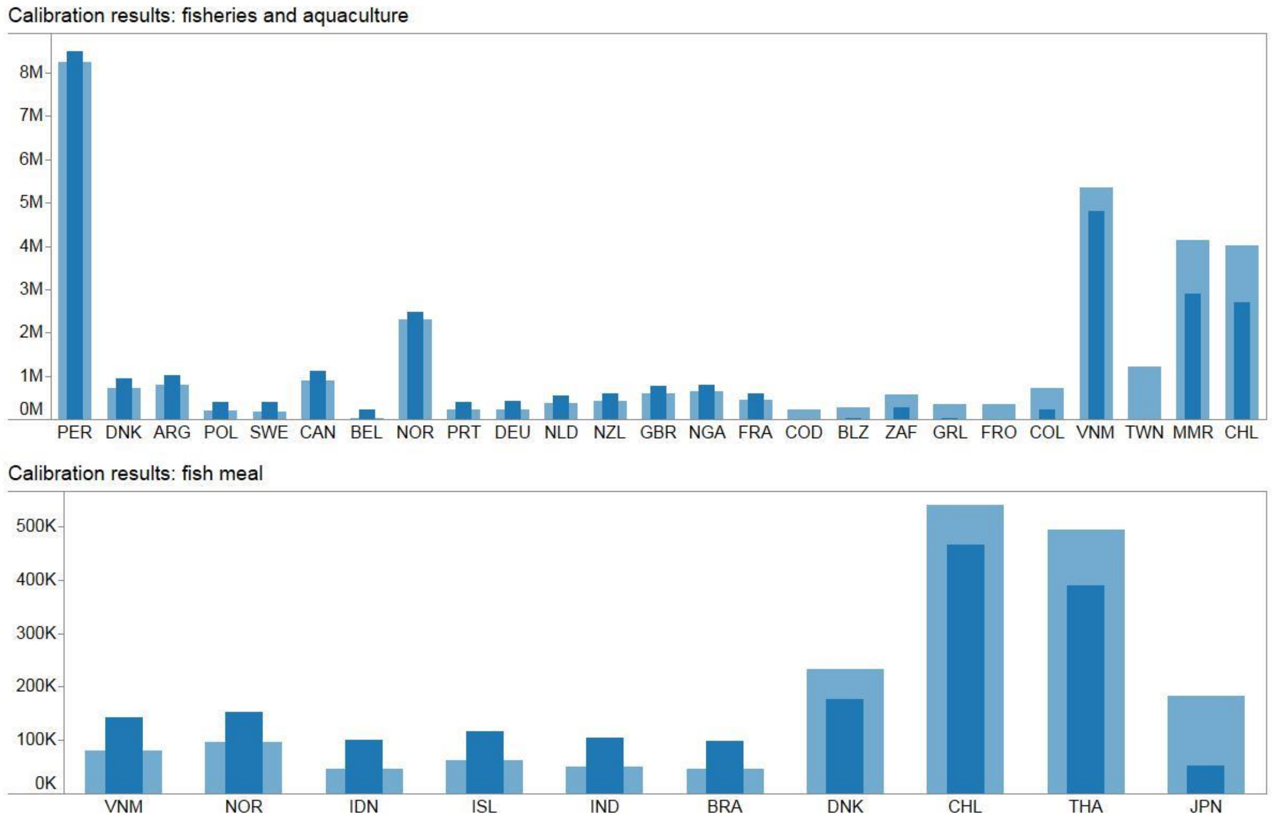

Table S1: Seafood consumption footprint (tonnes) and per capita consumption footprint (kg) for the aquaculture, capture fisheries and fishmeal sectors for the top 100 countries ranked according to their consumption for 2011.

Country	Country code	Total consumption footprint (tonnes)	Consumption footprint per capita (kg)			
			Total	Fisheries	Aquaculture	Fish meal
World		154 187 007	22.3	9.7	8.8	3.8
China	CHN	64 951 697	48.3	12.9	27.1	8.3
European Union	EU	13 051 508	25.7	14.4	5.7	5.6
Japan	JPN	7 412 488	58.0	35.3	14.6	8.1
Indonesia	IDN	7 297 806	30.1	15.5	9.6	5.1
United States	USA	7 143 636	22.9	13.1	5.3	4.5
India	IND	6 331 374	5.1	2.3	2.3	0.4
Korea, Republic of	KOR	3 907 340	78.5	48.9	21.7	7.8
Russian Federation	RUS	3 405 490	24.0	16.6	3.8	3.5
Viet Nam	VNM	3 257 070	37.1	11.6	16.5	9.0
Philippines	PHL	3 247 409	34.2	22.5	8.5	3.3
Bangladesh	BGD	3 007 999	20.0	8.4	9.7	1.8
Nigeria	NGA	2 971 053	18.3	12.0	4.4	1.8
Myanmar	MMR	2 897 091	59.9	44.7	11.8	3.4
France	FRA	2 391 316	36.5	22.4	8.1	6.0
Brazil	BRA	2 179 102	11.1	2.4	4.4	4.3
Spain	ESP	2 138 501	46.3	28.3	9.1	8.8
Egypt	EGY	1 878 771	22.8	3.7	13.2	5.8
Thailand	THA	1 785 733	25.7	9.8	8.6	7.4
Italy	ITA	1 715 292	28.2	15.7	7.0	5.5
Malaysia	MYS	1 691 515	58.6	35.6	14.4	8.6
Germany	DEU	1 375 334	16.8	9.1	3.9	3.9
United Kingdom	GBR	1 325 170	21.2	11.4	5.0	4.7
Mexico	MEX	1 320 667	11.5	6.9	1.7	2.9
Peru	PER	850 602	28.9	21.9	0.3	6.7
Canada	CAN	836 166	24.2	12.8	5.4	6.0
Ghana	GHA	680 875	27.3	23.5	2.6	1.2
Ukraine	UKR	675 578	14.8	9.0	2.6	3.2
Portugal	PRT	654 682	61.5	40.0	11.7	9.8
Turkey	TUR	651 694	8.8	3.6	2.0	3.3
Iran, Islamic Republic of	IRN	624 954	8.4	2.1	2.8	3.5
Australia	AUS	622 112	27.5	12.6	8.7	6.3
Poland	POL	564 598	14.8	7.4	3.0	4.4
Sri Lanka	LKA	553 529	26.5	20.6	3.6	2.3
Cambodia	KHM	518 864	36.3	29.5	4.4	2.3
Netherlands	NLD	461 804	27.7	14.6	5.0	8.1
Uganda	UGA	460 743	13.4	10.2	2.2	1.0

Morocco	MAR	447 683	13.9	12.5	0.3	1.1
Venezuela	VEN	414 051	14.1	8.9	1.7	3.6
Cameroon	CMR	386 995	19.3	15.9	2.0	1.5
Côte d'Ivoire	CIV	357 937	17.8	14.8	2.0	0.9
Pakistan	PAK	350 696	2.0	1.2	0.5	0.3
Angola	AGO	334 368	17.0	14.3	1.1	1.6
Sweden	SWE	332 778	35.2	19.4	8.3	7.5
Norway	NOR	329 787	66.6	26.2	16.2	24.2
Senegal	SEN	329 715	25.8	25.0	0.1	0.7
Belgium	BEL	310 421	28.2	14.5	7.1	6.6
South Africa	ZAF	310 029	6.1	3.5	1.1	1.5
Chile	CHL	309 374	17.9	7.1	3.4	7.4
Colombia	COL	301 637	6.4	3.8	0.9	1.7
Greece	GRC	290 579	25.7	6.4	8.9	10.5
United Republic of Tanzania	TZA	276 750	6.0	5.5	0.3	0.2
Saudi Arabia	SAU	251 624	9.0	3.9	2.3	2.8
Argentina	ARG	243 094	6.0	3.7	0.7	1.5
Korea, Democratic People's Republic of	PRK	235 150	9.6	6.3	2.6	0.7
United Arab Emirates	ARE	220 189	27.9	19.3	5.6	3.0
Finland	FIN	212 891	39.5	27.2	5.1	7.3
Mozambique	MOZ	209 178	8.7	8.1	0.1	0.5
Denmark	DNK	198 235	35.6	11.1	3.7	20.7
Sierra Leone	SLE	196 459	32.8	32.4	0.1	0.3
Kenya	KEN	189 982	4.6	3.8	0.5	0.3
Belarus	BLR	186 885	19.7	9.6	4.5	5.6
Israel	ISR	174 076	22.4	6.4	10.1	5.9
Romania	ROU	165 764	7.7	2.5	2.2	3.0
Switzerland	CHE	151 384	19.1	10.1	4.8	4.3
Algeria	DZA	151 173	4.2	2.8	0.6	0.8
Ecuador	ECU	146 490	10.0	2.3	2.4	5.4
Tunisia	TUN	146 064	13.7	8.2	2.5	3.0
Lithuania	LTU	143 951	44.9	31.5	6.1	7.3
Benin	BEN	133 701	14.7	12.8	1.0	0.8
Austria	AUT	125 884	15.0	8.4	2.8	3.8
Mali	MLI	119 235	7.5	7.0	0.1	0.4
New Zealand	NZL	118 051	26.8	17.2	6.5	3.2
Czech Republic	CZE	115 121	10.9	5.5	2.9	2.5
Congo	COG	114 828	27.7	21.6	3.2	3.0
Ireland	IRL	110 559	24.6	13.9	4.6	6.2
Lao People's Democratic Republic	LAO	108 550	17.3	0.8	12.5	3.9
Guinea	GIN	108 144	10.6	10.2	0.1	0.3
Burkina Faso	BFA	107 859	6.4	5.0	0.3	1.1
Dominican Republic	DOM	107 248	10.7	3.2	2.7	4.8
Madagascar	MDG	105 749	5.0	3.6	0.5	0.9
Iraq	IRQ	99 352	3.0	1.5	1.0	0.5

Croatia	HRV	94 050	21.3	13.0	4.0	4.3
Zambia	ZMB	91 339	6.8	5.3	0.6	0.8
Kazakhstan	KAZ	87 040	5.3	3.0	0.8	1.4
Oman	OMN	81 896	28.8	26.2	1.6	0.9
Malawi	MWI	78 281	5.1	4.0	0.2	0.9
Jamaica	JAM	72 772	26.9	14.6	5.5	6.8
Serbia	SRB	68 514	9.4	3.0	2.5	4.0
Cuba	CUB	66 671	5.9	1.4	2.6	1.9
Latvia	LVA	62 325	28.1	21.4	2.7	4.0
Hungary	HUN	61 500	6.2	1.2	2.2	2.8
Chad	TCD	60 745	5.3	5.2	0.0	0.1
Nepal	NPL	60 359	2.0	0.4	1.1	0.4
Yemen	YEM	60 266	2.4	1.2	0.5	0.7
Bulgaria	BGR	57 763	7.7	3.0	2.4	2.3
Lebanon	LBN	55 937	13.1	4.8	3.7	4.6
Moldova, Republic of	MDA	55 735	15.7	6.5	5.8	3.4
Gabon	GAB	55 243	36.0	29.5	3.7	2.8
Costa Rica	CRI	54 492	11.5	3.7	4.4	3.5
